# Relationship of Productivity to Species Richness in the Xinjiang Temperate Grassland

**DOI:** 10.1371/journal.pone.0154026

**Published:** 2016-04-21

**Authors:** Lili Liu, Junhui Cheng, Yunhua Liu, Jiandong Sheng

**Affiliations:** Xinjiang Key Laboratory of Soil and Plant Ecological Processes, College of Grassland and Environmental Sciences, Xinjiang Agricultural University, Urumqi, Xinjiang Uygur Autonomous Region, China; Tennessee State University, UNITED STATES

## Abstract

The relationship between species richness (SR) and aboveground net primary productivity (ANPP) is still a central and debated issue in community ecology. Previous studies have often emphasized the relationship of alpha diversity (number of species identity) to the mean ANPP with respect to the SR-ANPP relationship while neglecting the contribution of beta diversity (dissimilarity in species composition) to the mean ANPP and to the stability of ANPP (coefficient of ANPP: CV of ANPP). In this study, we used alpha and beta diversity, mean ANPP and the CV of ANPP collected from 159 sites and belonging to three vegetation types in the Xinjiang temperate grassland to first examine their trends along climatic factors and among different vegetation types and then test the relationship among alpha (beta) diversity and mean ANPP and the CV of ANPP. Our results showed that in the Xinjiang temperate grasslands, alpha diversity was positively and linearly correlated with MAP but unimodally correlated with MAT. Meanwhile, beta diversity was unimodally correlated with MAP but linearly correlated with MAT. Relative to desert steppe, meadow steppe and typical steppe had the highest alpha and beta diversity, respectively. Except for ANPP exhibiting a quadratic relationship with MAP, no significant relationship was found among ANPP, the CV of ANPP and climatic factors. ANPP and the CV of ANPP also exhibited no apparent patterns in variation among different vegetation types. Our results further showed that mean ANPP was closely associated with alpha diversity. Both linear and unimodal relationships were detected between alpha diversity and mean ANPP, but their particular form was texture-dependent. Meanwhile, the CV of ANPP was positively correlated with beta diversity. Our results indicated that in addition to incorporating alpha diversity and mean ANPP, incorporating beta diversity and the CV of ANPP could expand our understanding of the SR-ANPP relationship.

## Introduction

The relationship between species richness (SR) and aboveground net primary productivity (ANPP) has been a central issue in community ecology [1–3]. Despite the patterns and mechanisms of the SR-ANPP relationship having been widely discussed in most terrestrial ecosystems [4,5], the reported SR-ANPP relationship remains controversial, ranging from a unimodal to positively linear relationship to a neutral form in different ecosystems [3,6,7]. These varied SR-ANPP relationships have often been explained by differently varying patterns in alpha diversity (numbers of species identity in community) along the ANPP gradient [5], whereas the contribution of beta diversity (dissimilarity in species composition in the community) to the SR-ANPP relationship has been neglected. Some recent studies have suggested that in addition to alpha diversity, beta diversity also plays an important factor in regulating the SR-ANPP relationship because species richness is heterogeneously distributed in ecosystems [8], providing a new mechanism for understanding the SR-ANPP relationship. However, our knowledge about the contribution of alpha and beta diversity to ANPP is still limited, and more studies are needed.

In particular, the mechanisms of alpha and beta diversity in influencing the SR-ANPP relationship are different. For alpha diversity, if the peak diversity occurs at the intermediate level of ANPP, then a unimodal SR-ANPP relationship will be observed [5,9]. If the peak diversity appears at the highest level of ANPP, then a positively linear relationship is produced [6,10,11]. For beta diversity, a high dissimilarity in species composition often appears at a high level of ANPP [12], which in turn generates a positive and linear relationship between beta diversity and ANPP [13]. These findings suggest that for a given ecosystem, the observed SR-ANPP relationship is simultaneously determined by alpha and beta diversity. For example, alpha and beta diversity alone or together can result in a positive and linear SR-ANPP relationship (if one exists). Thus, separately exploring the relationship among alpha diversity, beta diversity and ANPP could expand our understanding of the mechanisms influencing the SR-ANPP relationship.

Generally, ecologists have often used mean ANPP to test its relationship with species richness [2,3,6] while ignoring the connection between species richness and the coefficient of variation (CV) of ANPP. The CV of ANPP is another important dimension for understanding the SR-ANPP relationship because ANPP also shifts with the change in species composition and environmental conditions [2,6]. In particular, the CV of ANPP reflects the degree of stability in ANPP [14–16]. A greater (smaller) CV of ANPP indicates that the community has a lower (higher) stability in ANPP [14–16]. Despite the importance of the CV of ANPP in understanding ecosystem stability, studies on the connection of the CV of ANPP to alpha and beta diversity are still lacking, which in turn limits our understanding of the SR-ANPP relationship.

At a regional scale, the SR-ANPP relationship is also influenced by environmental factors and vegetation types because alpha and beta diversity, the mean ANPP and the CV of ANPP are significantly regulated by environmental factors and vegetation types [6,10,17]. A recent study reported that the positive SR-ANPP relationship observed in the temperate grasslands of China stemmed from the parallel responding patterns of SR (alpha diversity) and ANPP along a climatic gradient [10]. These studies imply that to better understand the SR-ANPP relationship, the variations in alpha and beta diversity, mean ANPP and CV of ANPP and the relationships among these variables should be separately tested in each vegetation type.

Here, we used alpha and beta diversity, mean ANPP and the CV of ANPP measured in the Xinjiang temperate grasslands in northwest China, which cover a wide range of climatic gradients and include different vegetation types, to address the following three questions: (1) how does alpha and beta diversity, mean ANPP and the CV of ANPP in the Xinjiang temperate grassland vary along a climatic gradient and among different vegetation types? (2) What is the general relationship between alpha diversity and ANPP and the CV of ANPP in the Xinjiang temperate grasslands? (3) What is the general relationship between beta diversity and ANPP and the CV of ANPP in the Xinjiang temperate grasslands?

## Materials and Methods

### Study area

**Fig 1:**
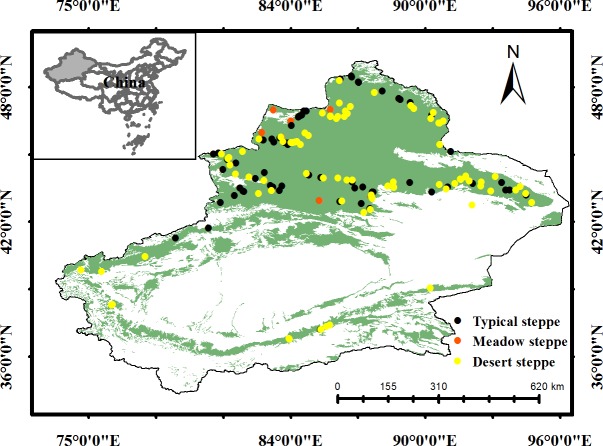
Location, study area and sites distribution of this study. The green color represented the place where was dominated by grassland.

This study was conducted in the Xinjiang Uygur Autonomous Region, located in the northwest part of China (Fig 1). The study area ranges from 34°25′N to 48°10′N in latitude and from 73°40′E to 96°18′E in longitude, covering an area of 16.6×10^6^ km^2^. Grassland is the predominant vegetation type and accounts for more than 34% of the total land area in XUAR, which is the third largest grassland in China [18]. In particular, the grasslands in this area belong to temperate grasslands in terms of climate, soil and vegetation composition [18].

The Xinjiang grasslands are characterized by a temperate continental climate. The mean annual precipitation (MAP) ranges from 10 to 550 mm, with 34% of precipitation occurring in the growing season (May-August). The mean annual temperature (MAT) ranges from 9 to 12°C, with the lowest mean monthly temperature in January (-20°C) and the highest in July (33°C). The major soil types in the Xinjiang grasslands are sandy soil, brown desert soil, brown calcic soil, frigid frozen soil and lithosol soil. The Xinjiang grasslands include nine vegetation types according to the variation in dominant species: alpine meadow (dominated by *Carex liparocarpos* and *Polygonum viviparum*), alpine steppe (dominated by *Stipa purpurea* and *Seriphidium rhodanthum*), lowland meadow (dominated by *Phragmites australis* and *Glycyrrhiza uralensis*), temperate steppe (dominated by *Stipa sareptana* and *Carex liparocarpos*), temperate desert (dominated by *Seriphidium terrae-albae* and *Ceratocarpus arenarius*), temperate desert-steppe (dominated by *Stipa caucasica* and *Seriphidium*), temperate steppe-desert (dominated by *Stipa Sareptana* and *Seriphidium heptapotamicum*), temperate mountain meadow (dominated by *Achillea millefolium* and *Poa pratensis*), and temperate meadow steppe (dominated by *Stipa purpurea* and *Carex spp*.) [18].

### Survey of alpha, beta diversity, mean values and CV of ANPP

We investigated alpha and beta diversity, mean ANPP and the CV of ANPP in the Xinjiang temperate grasslands in 2011, 2012 and 2013. A survey was conducted in the summers (July-August) of each year because ANPP reaches its peak value during this period [19]. In this study, only temperate meadow steppe (MS), temperate steppe (TS) and temperate desert steppe (DS) data were used for further analysis because their sample sizes satisfied statistical requirements and were proportional to their distribution area (Fig 1). Thus, a total of 159 field sites, which were evenly distributed in the Xinjiang temperate grasslands, were included in this study (Fig 1).

At each site, a 100×100 m sampling area was randomly selected. After recording the geographical coordinates, elevation and vegetation types, three or five 1×1 m quadrats were randomly chosen within this sampling area in each site. For each quadrat, we first recorded species names and the individual numbers for each species, and then we harvested the ANPP for each quadrat. We pre-dried the harvested ANPP in the field and then dried it to constant weight at 70°C in an oven in the laboratory of Xinjiang Agriculture University [10]. Based on these investigated data, we calculated the alpha and beta diversity, mean ANPP and CV of ANPP for each site. In particular, alpha diversity was expressed by the mean number of species appearing in all quadrats of each site. The beta diversity for each site was calculated as the proportion of unshared species among these investigated quadrats (one minus the Jaccard index). Specifically, beta diversity was calculated via following two steps. Firstly, for any two given quadrats (i.e. quadrats *i* and *j*, respectively), we calculated the proportion of unshared species between them using the equation: 1-a/(a+b+c), where a is the number of shared species between two quadrats, b is the number of species that appeared in quadrats *i* but not in *j* while c is the number of species that occurred in quadrat *j* but not in *i* [13,20]. This procedure was repeated to calculate the beta diversity between any two combined quadrats (3 or 10 combinations if a site had three or five quadrats, respectively). Then, for each site, the mean values of beta diversity was calculated and further used to explore their relationship with ANPP (above-ground net primary productivity)[13]. The mean ANPP was expressed by averaging the ANPP in all quadrats, whereas the CV of ANPP was obtained by dividing the standard deviation of ANPP by its mean value.

### Collection of climatic variables

Based on the long-term meteorological data (1961–2010) obtained from 756 evenly distributed climatic stations in China, we calculated the mean annual precipitation (MAP) and mean annual temperature (MAT) of each site in our study. In detail, MAP and MAT were calculated from a Geographic Information System-based multiple regression method which used latitude, longitude and elevation as predicators [21]. The interpolated climatic conditions among our surveyed sites changed differently, with MAP ranged from 33.4 to 398.2 mm while MAT ranged from 2.9 to 12.7 °C, which revealed an apparent climatic gradient in terms of MAP and MAT.

### Statistical analysis

To resolve our first question, we used a general linear model (GLM) to examine the relationship between MAP, MATand alpha and beta diversity, mean ANPP and the CV of ANPP separately [6]. For each examination, we fitted species diversity and ANPP as both a linear and quadratic function of the climatic factors. If the quadratic term did not statistically reduce the residuals (*a* = 0.05), then we dropped it from the model. The best-fitted model was selected using the Akaike Information Criterion (AIC) [22]. An unbalanced one-way ANOVA followed by LSD multiple comparisons was also used to test the variation of alpha and beta diversity, mean ANPP and the CV of ANPP among vegetation types [6].

To resolve our second and third questions, GLM was also used to examine whether a significant linear or quadratic relationship existed between alpha/beta diversity and the mean ANPP/CV of ANPP in each of vegetation type [10,23]. The best-fitting relationship between diversity and ANPP was also selected by AIC in this analysis. All statistical analysis were performed in R software(Vienna, Austria) [24].

## Results

### Variations of alpha, beta diversity, mean value and CV of ANPP along climatic gradient

**Fig 2:**
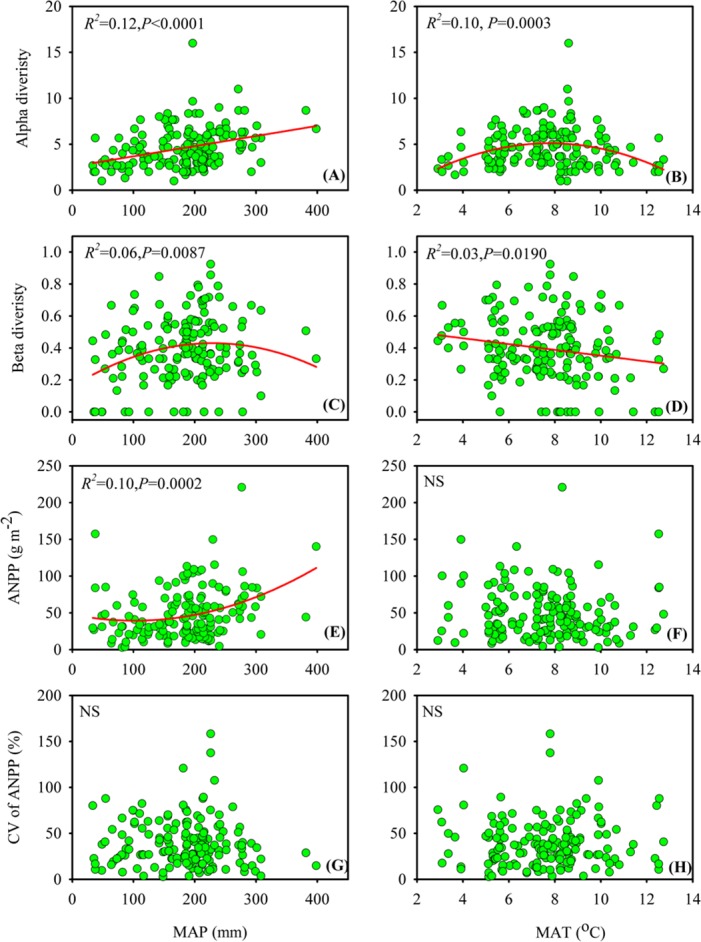
Relationship of alpha and beta diversity, ANPP and the CV of ANPP with mean annual precipitation (MAP) (A,C,E,G) and mean annual temperature (MAT) (B,D,F,H) in the Xinjiang temperate grasslands. NS indicates that no significant relationship was detected.

In the Xinjiang temperate grasslands, alpha and beta diversity, mean ANPP and the CV of ANPP responded differently to climatic factors at the site level (Fig 2). In particular, alpha diversity was positively and linearly correlated with MAP but exhibited a unimodal relationship with MAT (Fig 2A and 2B), indicating that peak alpha diversity occurred in sites with an intermediate level of MAT. However, beta diversity showed a unimodal relationship with MAP but negatively decreased with increasing MAT (Fig 2C and 2D), suggesting that peak beta diversity appeared in sites with an intermediate level of MAP. ANPP was only significantly correlated with MAP, with a tendency of decreasing with low levels of MAP but greatly increasing with high levels of MAP (Fig 2E). No significant relationship was detected between the CV of ANPP and MAP or MAT (Fig 2G and 2H).

### Variations of alpha, beta diversity, mean value and CV of ANPP among vegetation types

**Fig 3:**
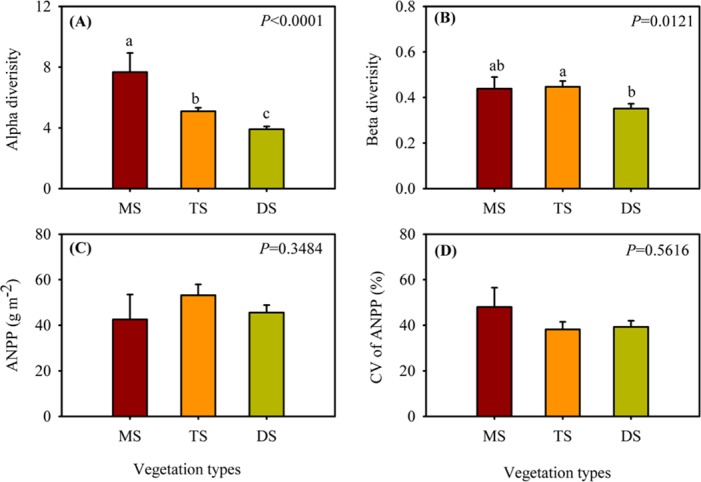
Variation in alpha (A) and beta diversity (B), ANPP (C) and the CV of ANPP (D) among meadow steppe (MS), typical steppe (TS) and desert steppe (DS) in the Xinjiang temperate grasslands.

Among vegetation types, alpha diversity was highest in meadow steppe but lowest in desert steppe, while beta diversity was highest in typical steppe but lowest in desert steppe (Fig 3A and 3B). On average, the alpha diversity in meadow steppe (7.7±1.3) was nearly twice as high as that in desert steppe (3.9±0.19) (Fig 3A), whereas beta diversity in typical steppe (0.44±0.05) was 1.29 times higher than that in desert steppe (0.35±0.02) (Fig 3B). No significant variation was detected in ANPP and the CV of ANPP among vegetation types. (Fig 3C and 3D). On average, ANPP in the Xinjiang temperate grasslands was 47.09 g m^-2^, and the CV of ANPP was 41.82%.

### Relationship of alpha diversity with mean value and CV of ANPP

**Fig 4:**
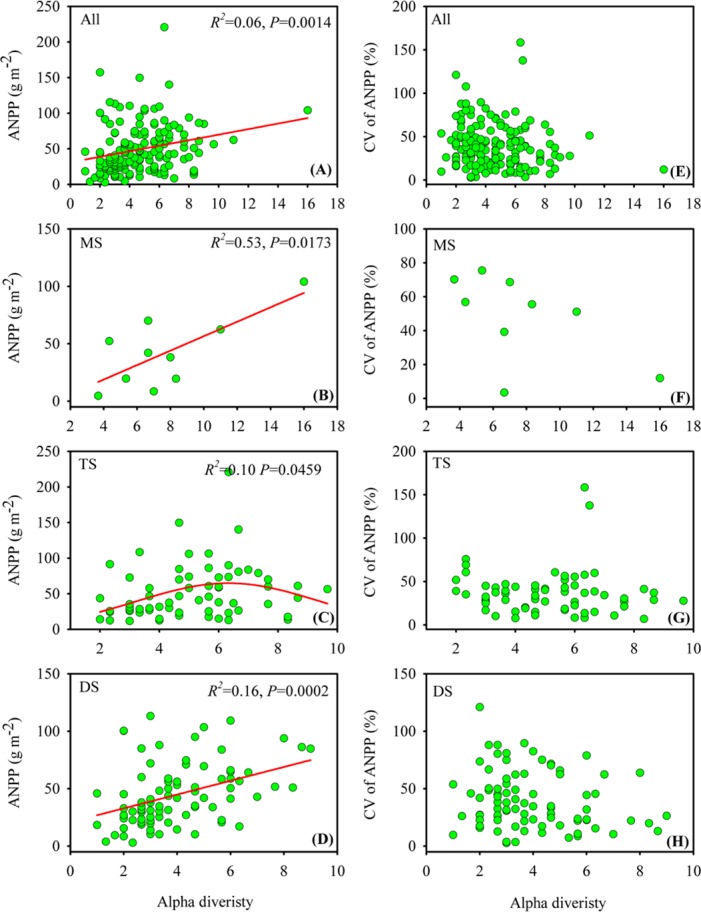
Relationship of alpha diversity with ANPP (A-D) and the CV of ANPP (E-H) in the Xinjiang temperate grasslands. MS, TS and DS are the abbreviations for meadow steppe, typical steppe and desert steppe, respectively. NS indicates that no significant relationship was detected.

In the Xinjiang temperate grasslands, alpha diversity was tightly correlated with ANPP but not with the CV of ANPP (Fig 4). When all sites were pooled into the analysis, alpha diversity was positively and linearly correlated with ANPP (Fig 4A). The positively linear relationship between ANPP and alpha diversity was still existed even the outlier (i.e. the dot on the most right location in Fig 4A) was removing from the data pool. When alpha diversity and ANPP were reclassified into vegetation types, the positive relationship between alpha diversity and ANPP was still detected in meadow steppe and desert steppe (Fig 4B and 4D), indicating that in meadow and desert steppe, high ANPP generally occurred in the sites that also had high alpha diversity. However, in typical steppe, their alpha diversity exhibited a significant unimodal relationship with ANPP (Fig 4C), suggesting that in typical steppe, high ANPP appeared in the sites that had an intermediate level of alpha diversity. Moreover, no significant relationship was found between alpha diversity and the CV of ANPP in either grasslands or each of these three vegetation types (Fig 4E–4H).

### Relationship of beta diversity with mean value and CV of ANPP

**Fig 5:**
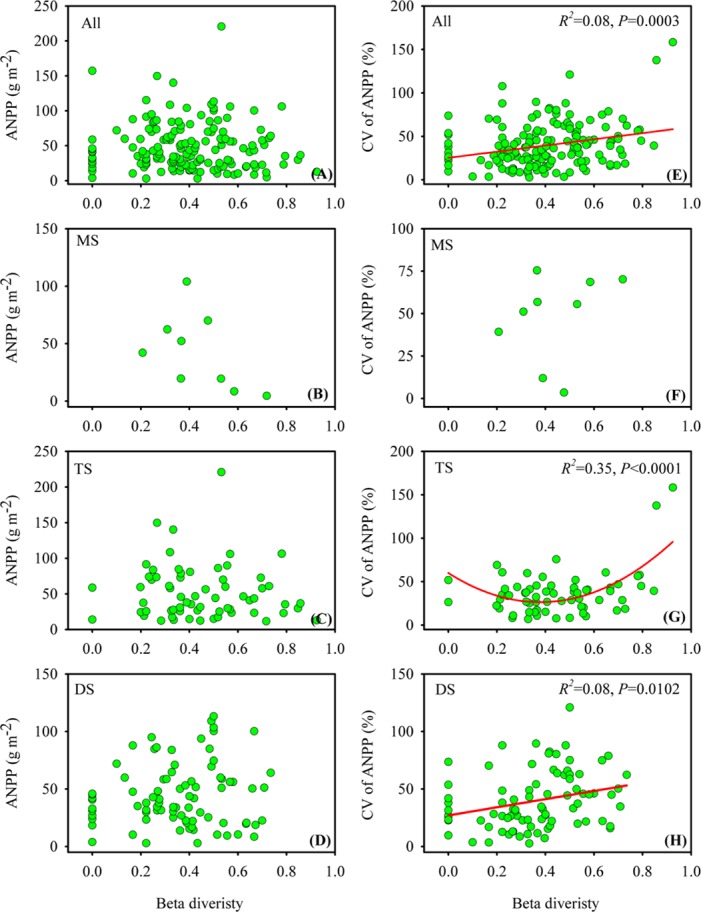
Relationship of beta diversity with ANPP (A-D) and the CV of ANPP (E-H) in the Xinjiang temperate grasslands. MS, TS and DS are the abbreviations for meadow steppe, typical steppe and desert steppe, respectively. NS indicates that no significant relationship was detected.

Our results revealed that in the Xinjiang temperate grasslands, beta diversity was significantly correlated with the CV of ANPP but not with ANPP (Fig 5). In all sites and desert steppe, a positive and linear relationship was found between beta diversity and the CV of ANPP (Fig 5E and 5H), demonstrating that the greater the dissimilarity in species composition, the more unstable the ANPP in the Xinjiang temperate grasslands and in desert steppe. Contrary to desert steppe, a significant quadratic relationship between beta diversity and the CV of ANPP was detected in typical steppe, with the tendency of decreasing in low beta diversity but increasing in high beta diversity (Fig 5G), indicating that in typical steppe, the high level of instability in ANPP occurred both in sites with less dissimilarity in species composition and in sites with large dissimilarity in species composition. In meadow steppe, however, no significant relationship was detected between beta diversity and the CV of ANPP (Fig 5F). Our results also revealed that beta diversity was unrelated to changes in ANPP in the Xinjiang temperate grasslands (Fig 5A–5D).

## Discussion

### Variations of alpha, beta diversity, mean value and CV of ANPP along climatic gradient

Our results showed that in the Xinjiang temperate grasslands, despite the influence on alpha and beta diversity by both MAP and MAT, alpha and beta diversity responded differently to MAP and MAT. We found that alpha diversity responded linearly to MAP but unimodally to MAT, which is consistent with previous studies conducted in Xinjiang and on a global scale [25,26]. These results were also in agreement with the prediction of the water-energy dynamics hypothesis, which suggests that in arid regions, species richness is positively correlated with water availability but negatively associated with energy availability [26,27]. Beside the effect of water and energy dynamics, the relationship between alpha diversity and MAP, MAT on our study was also influenced by the snow and snow melt water. Particularly, Xinjiang has a high snow depth (16.7–18.7 cm) and longer snow cover time (87.3–85.4 days a^-1^) relative to the other provinces in China [28]. It has reported that snow respectively accounted for more than 30% and 80% of annual precipitation in plain and mountains in our study area[29]. Moreover, the thawing period of snow in our study area was coincided with the period of species germination, indicating that snow melt water also had potential effects on species diversity distribution. Actually, snow melt water could influence species diversity via following two mechanisms: Firstly, snow melt water could indirectly decrease the air temperature during the processes of thaw, which may affect species germination time especially in the meadow steppe where was originally limited by temperature. Secondly, snow melt water will improve soil water availability, which can facilitate species diversity and plant growth in this arid regions[30]. In contrast to alpha diversity, we found that beta diversity responded unimodally to MAP but linearly to MAT, indicating that the highest dissimilarity in species composition appeared at the intermediate level of MAP.

The quadratic relationship between ANPP and MAP in our study was inconsistent with previous studies in the temperate grasslands in Inner Mongolia and in Kansas, which had reported a positive and linear correlation between ANPP and MAP. [6,17,31]. These different findings may be attributed to the difference in species composition between Xinjiang and Inner Mongolian grasslands because a previous study suggested that different grasses responded differently to water availability with respect to their aboveground biomass [32]. Moreover, the lack of a significant relationship between the CV of ANPP and MAP in our study was also inconsistent with one of these previous studies [17], suggesting that the stability of ANPP in the Xinjiang temperate grasslands is influenced by other potential factors (i.e., species composition) rather than by climatic factors.

### Variations of alpha, beta diversity, mean value and CV of ANPP among vegetation types

Our results also showed that in the Xinjiang temperate grasslands, alpha diversity in meadow steppe is significantly higher than that in typical steppe and desert steppe, which is consistent with the previous studies conducted in Inner Mongolian grasslands and in Tibetan grasslands [6,10]. However, the varying magnitude of alpha diversity among meadow, typical and desert steppes (7.7, 5.1, and 3.9, respectively) in our study is lower than that reported in Inner Mongolian grasslands (20.9, 14.0 and 11.6 for meadow, typical and desert steppes, respectively) and in Tibetan grasslands (17 and 8 for alpine meadow and alpine steppes, respectively). The low alpha diversity in our study could be explained by the relatively low aridity index (ratio of precipitation to evapotranspiration) in the Xinjiang temperate grasslands (0.01–0.10) because previous studies have confirmed that the plant species richness in arid and semi-arid regions in Northwest China is tightly determined by the aridity index [25,33].

The higher beta diversity in meadow and typical steppes observed in this study indicated that in the Xinjiang temperate grasslands, the species community is highly dissimilar in meadow and typical steppes, which is consistent with a previous study on Inner Mongolian grasslands, which reported that the highest species dissimilarity was observed under high MAP conditions (meadow steppe) [6]. Despite the significant variation in alpha and beta diversity observed, ANPP and the CV of ANPP exhibited no significant variation among meadow, typical and desert steppes in our study, suggesting that species diversity, ANPP and the CV of ANPP exhibited different patterns among vegetation types.

### Relationship of alpha diversity with mean value and CV of ANPP

Our results showed that the positively linear and unimodal relationship between species richness (alpha diversity) and ANPP occurred simultaneously in different vegetation types in the Xinjiang temperate grasslands. In particular, a positive and linear relationship was detected in meadow and desert steppes, but a unimodal relationship was found in typical steppe. This finding was inconsistent with a recent study that reported the unimodal relationship as a worldwide phenomenon in grasslands with respect to the relationship of species richness to ANPP [3]. Indeed, the texture-dependent relationship between alpha diversity and ANPP in our study was supported by a previous hypothesis suggesting that the relationship between species richness and ANPP changes from unimodal to positively linear as resource conditions shift from heterogeneity to homogeneity [5]. This is because in the resource-poor conditions in desert steppes, only few species with a tolerance strategy can survive and the strength of competition is weak [9,34], whereas in the resource-rich conditions in meadow steppes, the robust competition only allows a few dominant species with high competitive ability to exist [4]; in turn, the limited species number in desert and meadow steppes produce a positive relationship between alpha diversity and ANPP [5]. Because the resource conditions in typical steppe gradually transform from poor to rich, competition first increases with increasing ANPP, and then the competition under high ANPP conditions results in species loss, which in turn generates a unimodal relationship between alpha diversity and ANPP [5,35]. The lack of a significant relationship between alpha diversity and the CV of ANPP in our study indicated that the stability of ANPP was not determined by the number of species identified in the Xinjiang temperate grasslands.

### Relationship of beta diversity with mean value and CV of ANPP

Contrary to alpha diversity, we found that beta diversity was not significantly correlated with ANPP, which was consistent with a previous study conducted on Californian serpentine flora [13]. However, we further found that beta diversity was positively correlated with the CV of ANPP in each vegetation type except for meadow steppe. This is because in our study beta diversity was expressed by the proportion of unshared species among different quadrats, and high beta diversity thus indicates high dissimilarity in species composition within a site [23,36]. The positive relationship observed between beta diversity and the CV of ANPP may have resulted from the parallel response pattern of the dissimilarity in species composition and the variance in ANPP along the ANPP gradient [6].

## Conclusions

By examining variations in alpha and beta diversity, mean ANPP and the CV of APPP along climatic factors and among different vegetation types, our study showed that in the Xinjiang temperate grasslands, alpha and beta diversity were closely associated with both MAP and MAT and were highest in meadow and typical steppes relative to desert steppe. Except for ANPP exhibiting a quadratic relationship with MAP, no significant relationship was found between ANPP, the CV of ANPP and climatic factors. ANPP and the CV of ANPP also exhibited no apparent patterns in variation among different vegetation types. Based on testing the relationship between species diversity and ANPP, our results further showed that alpha diversity was tightly correlated with mean ANPP, whereas beta diversity was significantly associated with the CV of ANPP. Our results demonstrated that a positive linear and unimodal relationship appeared simultaneously in different vegetation types in the Xinjiang temperate grasslands.
